# Flow Reduction in a Pesticide-Exposed Stream Mesocosm Affects Emerging Aquatic Insects and Alters Riparian Spider Communities

**DOI:** 10.1007/s00244-025-01146-5

**Published:** 2025-08-15

**Authors:** Collins Ogbeide, Alessandro Manfrin, Gemma Burgazzi, Florian Burgis, Anja Knäbel, Sebastian Pietz, Nina Röder, Alexis Pieter Roodt, Verena C. Schreiner, Klaus Schwenk, Mirco Bundschuh, Ralf Schulz

**Affiliations:** 1 iES Landau, Institute for Environmental Sciences, RPTU Kaiserslautern-Landau, Fortstrasse 7, 76829 Landau, Germany; 2 Eußerthal Ecosystem Research Station (EERES), RPTU Kaiserslautern-Landau, Birkenthalstraße 13, 76857 Eußerthal, Germany; 3 Faculty of Biology, University of Duisburg-Essen, Universitätsstrasse 2, 45141 Essen, Germany; 4 Research Center One Health Ruhr, University Alliance Ruhr, Universitätsstrasse 2, 45141 Essen, Germany; 5 ALPSTREAM Group, Department of Life Science and System Biology, University of Turin, Turin, Italy

## Abstract

Water scarcity can intensify streamflow reduction, alter hydromorphology, increase chemical pollution, and disrupt resource exchange between aquatic and terrestrial ecosystems. However, the effects of streamflow reduction on pesticide concentrations in sediment, and how these changes influence aquatic insect emergence and riparian spider communities, remain poorly understood. We conducted a 39-day mesocosm experiment in Southwest Germany using 12 artificial stream mesocosm with adjacent riparian areas, randomly assigned to low-flow (0.4 L s^−1^) treatment or control (1 L s^−1^) to simulate flow alteration. We sampled water daily (47 days), sediment (weeks 4 and 6), emerging insects (weekly for 5 weeks), and riparian spiders (week 6). Our results show that under low-flow conditions, the mean sediment pesticide concentrations increased slightly stronger from week 4 to week 6, compared to the control. However, the high variability within treatments resulted in no significant differences between the treatments. Low flow caused a 2.9-fold reduction in the total abundance of Ephemeroptera, Plecoptera, and Trichoptera (EPT), while total emerging insects (abundance and biomass) and spider abundance were not affected significantly. However, the web-building spider *Tetragnatha* sp. was 2.6-fold less abundant under low-flow conditions, likely due to vegetation–habitat-related responses and reduced EPT abundance, while the ground-hunting Lycosidae were unaffected, owing to their active hunting behavior and generalist strategy. These findings highlight the need to consider cross-ecosystem effects when assessing the ecological impacts of hydrological alterations in contaminated aquatic ecosystems.

**Supplementary Information:**

The online version contains supplementary material available at 10.1007/s00244-025-01146-5.

## Introduction

As global change and anthropogenic activities continue to impact aquatic ecosystems (Vörösmarty et al. 2000; Schulz et al. 2024), it is estimated that more than 50% of the global river network by length is experiencing at least one day of flow cessation annually (Messager et al. 2021). Indeed, increasing temperatures and evaporation, especially during summer in combination with reduced precipitation and ground water influx, can drastically reduce water levels (Smakhtin 2001; Boulton and Hancock 2006; Schneider et al. 2013). These low-flow periods, in turn, often coincide with increasing human water demands for agriculture and domestic needs (Dewson et al. 2007a). As a result, such hydrological alterations in rivers can affect both abiotic and biotic processes, including oxygen and temperature dynamics, contaminant fate, litter decomposition, and structure of riverine communities (Rolls et al. 2012; Northington and Webster 2017). These effects can cascade to connected ecosystems, through the disruption in the flow of nutritional aquatic subsidies, such as emerging aquatic insects (Twining et al. 2016). The flows link aquatic and terrestrial ecosystems and serve as a vital food source for riparian consumers, with spider being among the primary recipients (Rolls et al. 2012; Schulz et al. 2015).

Pesticides, widely used in agriculture and introduced into aquatic ecosystems through both point and diffuse sources, are a significant source of contamination in freshwater ecosystems worldwide (Neumann et al. 2002; Stehle and Schulz 2015). Indeed, they can be a major driver in the decline of nontarget macroinvertebrate communities in streams (Liess et al. 2021). Under low-flow conditions, pesticide concentrations in streams can increase due to reduced dilution, increased deposition of pesticide-laden particles, and prolonged hydraulic residence time (Gaullier et al. 2020; Graham et al. 2024). These factors contribute to sustained exposure for nontarget communities inhabiting the water column and sediment (DeLorenzo et al. 2001; Schäfer et al. 2007). Consequently, pesticides can biomagnify through the flow of aquatic subsidies via emerging insects, transferring higher pesticide loads to terrestrial consumers and thereby impacting higher trophic levels and altering broader food web dynamics (Kraus 2019; Bundschuh et al. 2022; Roodt et al. 2023). Therefore, assessing cumulative pesticide concentrations in stream sediment under low-flow conditions is essential for understanding water quality and its potential impacts on linked aquatic–terrestrial ecosystems (Schulz et al. 2015).

Low-flow conditions lead to changes in stream hydrological parameters and microhabitats, including reductions in wetted width, depth, and mean velocity, as well as increases in temperature, decreased oxygenation, and nutrient concentrations (Dewson et al. 2007b). These changes can lead to either an increase or a decrease in macroinvertebrate abundance, biomass, and taxa richness, depending on the extent of flow reduction (Dewson et al. 2007a; Miller et al. 2007; Walters and Post 2011). For instance, Dewson et al. (2007c) observed that a 98% short-term reduction in water discharge led to increased local macroinvertebrate abundance, attributed to the accumulation of individuals in the reduced wetted habitat. In contrast, Walters & Post (2011) found that total macroinvertebrate abundance decreased in riffle habitats while remaining unchanged in pools, and reported a sharp overall decline in aquatic insect biomass under reduced flow, with no corresponding shift in family richness. Furthermore, these responses are taxa-specific, with low flow under altered abiotic factors causing a decrease in abundance of sensitive species such as Ephemeroptera, Plecoptera, and Trichoptera (EPT) (Dewson et al. 2007c; Miller et al. 2007; Carlisle et al. 2014). Upon completing their aquatic life stages, these macroinvertebrates, along with other aquatic insects such as dipterans, emerge as terrestrial flying adults. In doing so, they serve as a high-quality prey source for terrestrial consumers such as spiders (Twining et al. 2016; McKie et al. 2018).

Riparian spiders, especially those constructing horizontal orb webs on riparian vegetations, are among the first recipients of aquatic subsidy, partially relying on aquatic insects as their prey (Paetzold et al. 2005; Wieczorek et al. 2015; Bollinger et al. 2023). Consequently, changes in the abundance of emerging insects as a subsidy of terrestrial food webs can influence cross-ecosystem interactions (Kato et al. 2003; Rolls et al. 2012). For example, Greenwood and McIntosh (2010) reported that reduced aquatic insect subsidies under low-flow conditions led to a decrease in the biomass and size–class structure of the spider *Dolomedes aquaticus*. Additionally, Kato et al. (2003) found that the temporal dynamics of aquatic insect flux and the degree of spider specialization were key determining factors for distribution patterns. Taken together, the impacts of low-flow conditions on the fate of pesticides in stream sediments, aquatic insect emergence, and the potential cascading effects on terrestrial consumers in adjacent ecosystems remain poorly understood.

In this study, we used 12 mesocosm units, each consisting of an artificial stream (flume) and its adjacent riparian area, to assess the effects of low flow on flume sediment pesticide concentrations, the abundance and biomass of emerging aquatic insects, and the indirect effects on riparian spider abundance. We hypothesized that under low-flow conditions: (I) pesticide concentrations in stream sediments would be elevated due to decreased flow velocity, which increases residence time and enhances the sedimentation of suspended particles, (II) the abundance and biomass of emerging aquatic insects, and the abundance of EPT taxa, which are considered more sensitive, would decrease due to altered physicochemical conditions and the expected increase in pesticide concentrations in sediments, and (III) riparian spider abundance would decline due to reduced availability of aquatic subsidy and changes in habitat conditions. We expect these effects to be more pronounced in spider species that are specialized in preying on aquatic emerging insects and prefer riparian habitats (Kato et al. 2003; Paetzold et al. 2005).

## Material and Methods

### Study Site

**Fig. 1:**
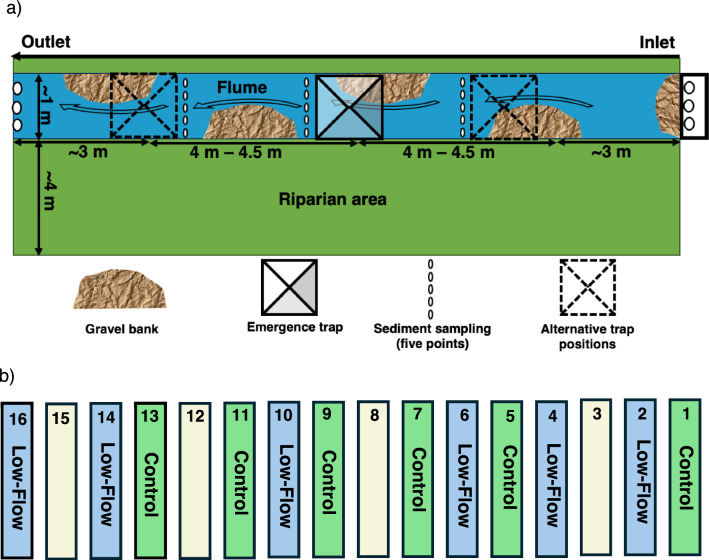
Experimental setup of RSM. **a** Schematic of an RSM unit, showing the flume and adjacent riparian area. The emergence trap was placed partially on the gravel bank to reflect habitat heterogeneity and positioned at either the inlet, middle, or outlet section within each flume. Solid outlines indicate the trap position in each flume, while dashed outline represent alternative positions used in other flumes, illustrating placement variation across flumes. **b** Diagram of random allocation of flume to individual treatments across the twelve RSM units; units 3, 8, 12, and 15 were intentionally excluded from the experiment

The experiment was conducted in 12 of the 16 replicated mesocosm units at the Riparian Stream Mesocosm (RSM) facility, located in Southwestern Germany, near Landau (49°12′03.9″ N 8°08′21.6″ E) (Manfrin et al. 2023; Rovelli et al. 2024). Each mesocosm unit includes a 15 m × 1 m flume fed in flow-through mode by water from the adjacent river Queich, as well as an adjacent 15 m × 4 m riparian habitat (Manfrin et al. 2023). The Queich flows through both urban and rural areas characterized by vineyards and intensive agriculture. Consequently, the water from the Queich contains various contaminants (Bereswill et al. 2012; Roodt et al. 2023). Each flume was built with side walls and a bottom made of impervious clay to prevent lateral and groundwater inflow (Rovelli et al. 2024). In each flume, we placed four gravel banks, composed of pebbles (16–32 mm) and cobbles (50–120 mm), alternating between the left and right flume banks at 1.75-m intervals (Fig. 1a) to enhance flow heterogeneity and thus have a more diverse habitat structure (Hose et al. 2007).

### Experimental Setup

The initial wetting of the flumes began on May 3, 2021, with a controlled pump discharge rate of 1 L s^−1^ (BADU® Eco Flex, Q Max 40 m^3^ h^−1^, SPECK PUMPEN GmbH, Neunkirchen, Germany). To prevent larger debris from entering the pipe system, the water inlet in the Queich was enclosed in a perforated metal sheet (6 mm in diameter), allowing for continuous passive colonization of the flumes by macroinvertebrates. Hydrologically, a gradient of flow velocities was observed in the flumes, with 0.25 ± 0.03 m s^−1^ (mean ± standard error) near the inlet and 0.11 ± 0.01 m s^−1^ adjacent to the outlet, along with variation in the water depth (Fig. 1a). To support the establishment of a diverse riverine freshwater macroinvertebrate community, we supplemented passive macroinvertebrate colonization by first deploying 48 coarse leaf bags (15 × 15 cm, 8 mm mesh size), each containing five alder (*Alnus glutinosa*) leaves, in the river Queich serving as habitat for macroinvertebrates. After one week, the leaf bags were retrieved and three were randomly allocated to each flume. Additionally, on May 18, 2021, macroinvertebrates and organic materials for active seeding were collected from the river Queich using a kick-net (0.5 mm mesh size) for 45 min. The collected materials were then pooled and evenly distributed near the inlet section of each flume.

The riparian habitat was seeded with Regioseed wetland seed mixture of 70% grasses, 30% herbs and legumes (Saatgut RegioZert®) on May 3, 2021, and watered regularly with tap water until the vegetation reached maturity (Fig. 1a). The riparian area was passively colonized from the surrounding grassland arthropods community. Finally, each unit was enclosed in a tent (15 m × 6 m, with a max height of 2 m), made of 1-mm mesh gauze, on June 10–11, 2021, to prevent the exchange of flying insects during the treatment phase of the experiment (Manfrin et al. 2023).

### Experimental Design

At the start of the experiment, six control flumes were maintained at the normal discharge rate of 1 L s^−1^. The experiment ran for five weeks and four days and was initiated on June 15, 2021, by reducing the discharge rate (low-flow treatment) to 0.4 L s^−1^ in six randomly selected flumes (Fig. 1b). This reduction resulted in an average discharge decrease of 60% (Rovelli et al. 2024). To ensure consistent environmental conditions and minimize potential confounding factors among the RSM flumes, physical and chemical water parameters (flow velocity, depth, temperature, dissolved oxygen, and conductivity measured with WTW probes, Weilheim, Germany) were measured three times in both control and low-flow flumes during summer 2021 (Table S1). The pH, conductivity, dissolved oxygen, and temperature were continuously measured using a logger (SENECT®, Landau, Germany) adjacent to the outflow to monitor temporal variations throughout the experimental period (Fig. S1).

### Pesticide Analysis of Water and Flume Sediment

Water samples were collected daily from June 7, 2021, to July 23, 2021, at the pumping station on the river Queich, which supplies the flumes, to characterize the pesticide input into the system. Additionally, composite sediment samples were collected from the upper 2 cm of the sediment bed at five points across the width in each of the three sections (inlet, middle, and outlet) of each flume (Fig. 1a) and pooled across all sections to form one sample per flume. The sediment samples were collected twice during the experiment, namely in the fourth and sixth weeks after the start of the experiment. All samples were stored at − 20 °C until analysis.

Water samples were analyzed using direct injection, while sediment samples were prepared and analyzed as described in Roodt et al. (2023). Briefly, stored frozen sediment samples were freeze-dried and sieved using a 1 mm mesh. A 5 g subsample was then weighed into a 50-mL Falcon tube from each sample. As a procedural internal standard, 50 µL of a solution containing pirimicarb-D6, thiacloprid-D4, and thiamethoxam-D3 in acetone was added to each sample to achieve a final concentration of 2 µg kg^−1^ in the measured extract. All internal standards were obtained from Restek (Bad Homburg, Germany).

The extraction of sediment samples was adapted from Bakanov et al. (2023). In this regard, 5 g of ammonium formate (reagent grade, Sigma-Aldrich, St Louis MO, USA) was added to each extraction tube, followed by 10 mL of acetonitrile (HPLC grade, Honeywell, Seelze, Germany) containing 2.5% formic acid (≥ 99%, VWR International, Leuven, Belgium). The samples were then shaken for 60 min using an overhead shaker. The resulting extract was centrifuged for 6 min at 1700 × g (relative centrifugal force, RCF), and the supernatant was filtered through a 0.2 µm polytetrafluoroethylene (PTFE) filter (17 mm, hydrophobic, HPLC grade; BGB Analytik, Lörrach, Germany) prior to pesticide analysis.

For the analysis, buffered mobile phases were prepared, consisting of methanol (MeOH, LC–MS grade, Honeywell, Seelze, Germany), water (LC–MS grade, Merck, Darmstadt, Germany), formic acid (≥ 99%, VWR International, Leuven, Belgium), and NH_4_HCO_2_ (≥ 99%, Sigma-Aldrich, St Louis, MO, USA). Subsequently, the concentrations of 96 current-use pesticides (Table S2, 36 fungicides; 36 herbicides, and 24 insecticides) were quantified in both water and sediment extracts using an HPLC–ESI–MS/MS system (Agilent 1290 Infinity II LC coupled to a 6495C triple quadrupole MS/MS, Agilent Technologies, Santa Clara, CA, USA). Quantification was performed using matrix-matched external calibration: for water, an 11-point calibration series ranging from 0.0003 to 2 µg L^−1^ was prepared in triplicate using MS-grade water along with solvent blanks; and for sediment, an 8-point calibration series ranging from 0.025 to 100 µg L^−1^ was used. The limits of quantification (LOQs) for water samples (µg L^−1^), as well as the LOQs and limits of detection (LODs) for sediment samples (µg kg^−1^), are presented in Table S2. Instrument parameters used for the HPLC–ESI–MS/MS analysis are summarized in Table S8. A detailed list of multiple reaction monitoring (MRM) transitions and associated collision energies (CE) used for the targeted MS/MS analysis of all analytes and internal standards is provided in Table S9 and applies to both water and sediment samples.

### Emerging Insect Sampling, Processing and Identification

Pyramidal emergence traps (basal area: 1 m^2^) with external collection bottles were used to sample emerging aquatic insects. In each flume, a single trap was placed either at the inlet, middle, or outlet flume section (Fig. 1a). A collection bottle, attached to the top of the trap, was filled with a trapping liquid consisting of water, propylene glycol, and soap (Cadmus et al. 2016). The collection bottles were emptied and replaced every seven days over a sampling period of five weeks, from June 15, 2021, to July 20, 2021. All emerging insects from each sample were sorted into four size categories (A–D) based on morphological characteristics such as body length and shape (Table S3). During sorting, individuals belonging to the orders EPT, as well as other non-dipteran taxa, were identified and counted by size category under a stereomicroscope (Zeiss Stemi 2000-C, 6.5× –50×) following Chinery et al. (2012). The order Diptera, dominated by the family Chironomidae, was assigned to size categories but not counted. Concurrently, a random subset of 59 samples (spanning all timepoints and size categories) was fully enumerated, including dipterans, to calibrate the dry mass–abundance relationship (cf. Sabo et al. 2002). After sorting, all 262 samples were rinsed in ethanol to remove the trap preservative, dried at 60 °C for at least 48 h, and weighed to the nearest 0.01 mg. For the remaining 203 samples (those not fully enumerated), abundance was estimated by dividing each sample’s total dry mass by the mean individual dry mass for the corresponding size category. Finally, both abundance and biomass were standardized to individuals per trap area (ind. m^−2^) and milligrams per trap area (mg m^−2^) per deployment day, respectively.

### Spider Sampling, Processing, and Identification

During the final four days of the 39-day experiment, we hand-collected long-jawed orb weavers (*Tetragnatha* sp.) from riparian vegetation and vegetation overhanging the water surface within each mesocosm, thoroughly inspecting each area until no further individuals were observed. They were counted, visually sorted to the genus level onsite, and subsequently stored at − 80 °C. Additionally, at the end of the experiment, ground-dwelling wolf spiders (Lycosidae) were collected across the entire riparian area of each mesocosm with suction sampling conducted in both the upstream and downstream sections using a modified leaf blower (petrol suction shredder SH 86, Stihl, Waiblingen, Germany) (Kormann et al. 2015) with 12 suction pulses over four minutes per section. The Lycosidae were counted and identified morphologically to the genus level using a taxonomic key (Roberts 1996) under a stereomicroscope (Leica M80 at 7.5×–60×), and stored at − 80 °C. The *Tetragnatha* sp. and Lycosidae were selected as representative riparian spider taxa based on their ecological traits, such as habitat specialization and foraging strategies (Roberts 1996; Graham et al. 2003).

### Data Analysis

To assess the effect of low-flow treatment on the flume sediment pesticide concentrations, we fitted a generalized linear mixed model (GLMM) using the glmmTMB package (version 1.1.10, Brooks et al. 2017). The model included sediment pesticide concentrations (µg kg^−1^) as response variables, with treatments (categorical: low flow, control, i.e., normal flow) and sampling weeks (categorical: week 4 and 6), as well as their interaction, as fixed factors. Flume ID was included as a random factor to account for repeated sampling event within each flume. The nonsignificant interaction term was dropped to reduce model complexity (Zuur et al. 2009), based on the results of the Type II ANOVA function from the car package (version 3.1.3, Fox & Weisberg 2019). The model was specified with a gamma distribution and a log link function, as the response variable was continuous, right-skewed, and nonnegative (Zuur et al. 2009). If serial autocorrelation was detected in the residuals of the initial model using the autocorrelation function (ACF) from the stats package (R Core Team 2025), we revised the model to include an autoregressive order 1 (AR-1) covariance structure. This adjustment addressed potential temporal pseudo-replication across sampling weeks within each flume. Additionally, since the residuals of the model were not normally distributed, we applied an inverse square root transformation to the sediment concentrations (µg kg^−1^) response variable. We applied the same model specification and family (Gamma distribution with a log link function) to the abundance and biomass of emerging aquatic insects, using treatments (categorical: low flow, control), sampling weeks (categorical: weeks 1–5), and trap position (categorical: inlet, middle, outlet) as fixed factors. The nonsignificant interaction term was dropped to reduce model complexity (Zuur et al. 2009). Additionally, to assess the effect of low-flow treatment on EPT abundance, we fitted a GLMM with EPT abundance as response variable, using a zero-inflated negative binomial family (nbinom2) with a log link. The model accounted for the interaction between week and position (categorical: inlet, middle, outlet), and for temporal autocorrelation within flumes over weeks, to address repeated measures and overdispersion. Finally, to assess the response of spider abundance to low flow, we fitted a GLMM with spider abundance as the response variable, including treatments (categorical: low flow, control), spider taxa (categorical: Tetragnatha and Lycosidae), and their interaction term as fixed factor, with Flume ID as random factor. A Poisson distribution with a log link function was used, as the response variable was count-based and nonnegative (Zuur et al. 2009).

To ensure model assumptions of normality and homogeneity of variance, we analyzed the model residuals using the simulateResiduals function of the DHARMa package (version 0.4.7; Hartig 2024) and examined QQ plots and residuals vs. fitted value plots for graphical visualizations. To examine differences between levels of the fixed effects, we conducted post hoc pairwise comparisons using the estimated marginal means (emmeans) package (version 1.10.6, Lenth 2024) for both significant interactions and main effects. To control the false discovery rate due to multiple testing, we applied Bonferroni-adjusted p-values for correction for the pairwise comparisons (version 1.10.6, Lenth 2024). Data wrangling and visualization were performed with tidyverse (version 2.0.0, Wickham et al. 2019) and rstatix (version 0.7.2, Kassambara 2023). All statistical analyses and visualizations were conducted in R (version 4.4.3 for macOS, R Core Team 2025). The code and all data are available on a GitHub repository: https://github.com/colinog/pollution_flow.

## Result and Discussion

### Pesticide Concentrations in Water Supplying the RSM

**Fig. 2:**
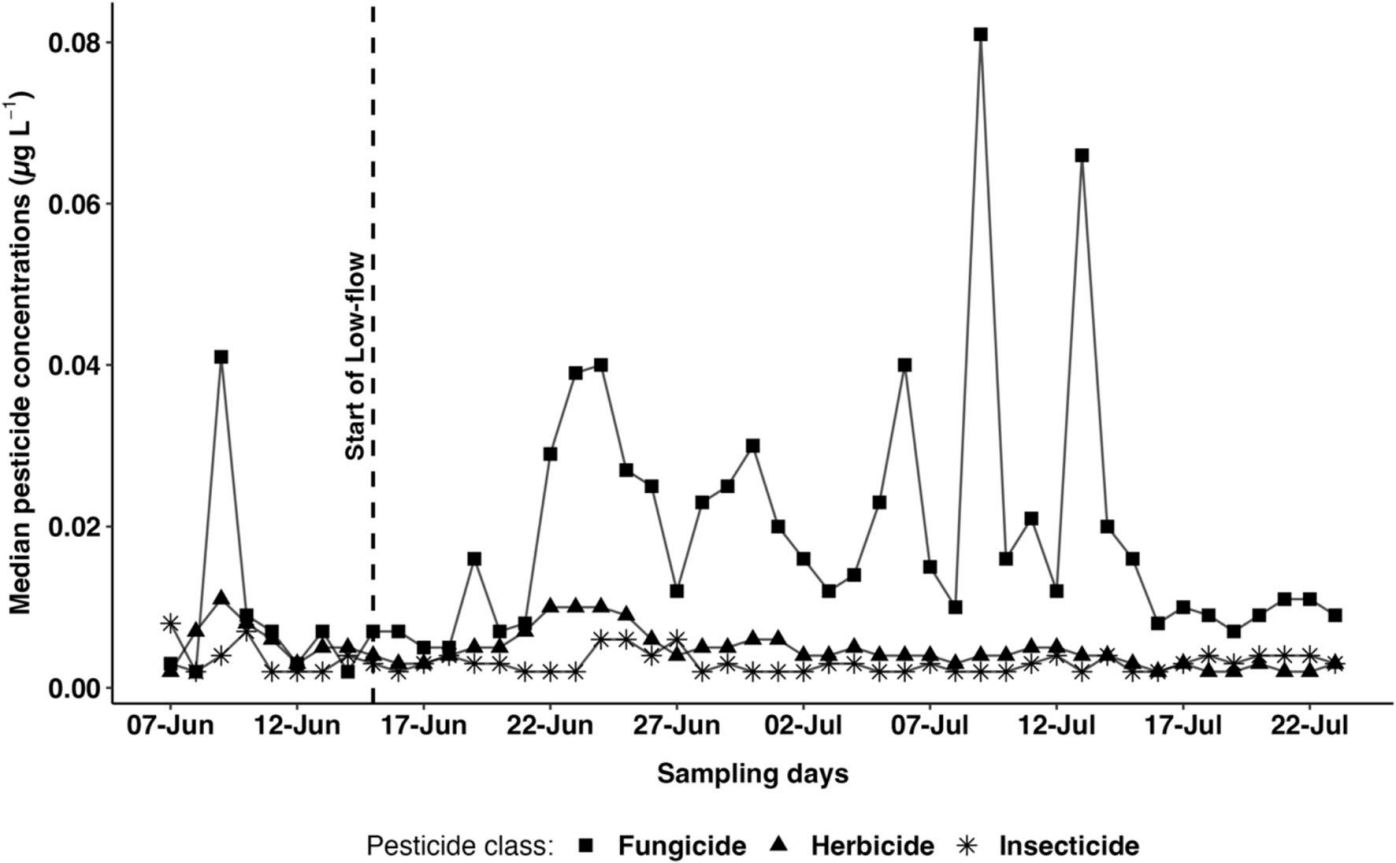
Time series plot of the median daily pesticide concentrations in water supplying the RSM. Rectangles represent fungicides, triangles represent herbicides, and asterisks represent insecticides. The vertical line “start of low-flow” indicates the beginning of the treatment phase in the flumes

Of the 96 pesticides analyzed (Table S2), 41 were detected at concentrations above the LOQs in daily sampled river water, providing a baseline of pesticide concentrations entering each flume. Among these pesticides, fungicides contributed the most (51%), followed by herbicides (29%), and insecticides (20%). Furthermore, the median concentration of fungicide, compared to other pesticide classes, fluctuated the most throughout the experimental period (Fig. 2, Table S4). The sum pesticide concentrations ranged from 0.001 to 1.68 µg L^−1^ in the water column suggesting a high daily fluctuation (Fig. 2, Table S4). The pattern of pesticide detection reflects the predominant land use in the river Queich catchment, which is primarily viticulture (Bereswill et al. 2012; Fernández et al. 2015). Specifically, fungicides commonly used in vineyards such as azoxystrobin, dimethomorph, metalaxyl, and boscalid were among the most frequently detected (Bereswill et al. 2012).

### Pesticide Concentrations in Flume Sediment Under Control and Low-Flow Conditions

**Fig. 3:**
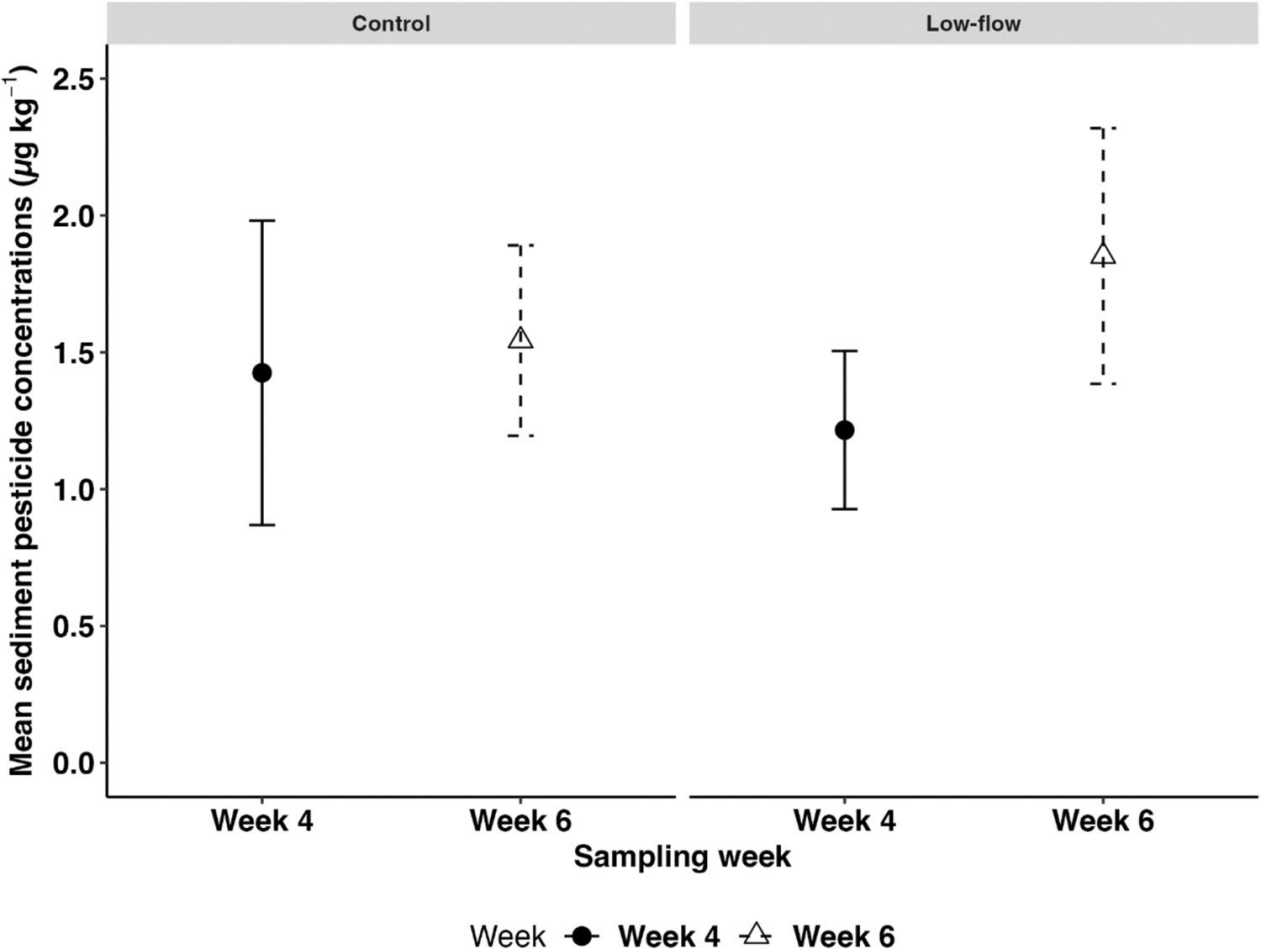
Mean sediment pesticide concentrations (n = 6) in control and low-flow treatments across two sampling weeks, with error bars representing the 95% confidence interval (CI). Circles (solid lines) represent week 4 samples, while triangles (dashed lines) represent week 6 samples in both the control and low-flow treatment

The concentration of pesticides in flume sediments did not differ between low-flow treatment and the control (GLMM: Estimate = − 0.0031, *p* = 0.954, Table S6, Fig. 3), which contradicts hypothesis I. We also did not observe significant temporal differences between weeks (GLMM: Estimate = − 0.080, *p* = 0.140, Table S6), likely due to high variability within the treatments. Nonetheless, the mean sediment pesticide concentrations increased by 52% in week 6 (mean = 1.85 µg kg^−1^ 95% CI ± 0.47) compared to week 4 (1.22 ± 0.29 µg kg^−1^) under the low-flow treatment (Fig. 3). In contrast, the control showed an 8.3% increase in week 6 (1.54 ± 0.35 µg kg^−1^) compared to week 4 (1.43 ± 0.56 µg kg^−1^). The increase in pesticide concentration in the low-flow treatment could be attributed to the accumulation of pesticide over time, driven by varying concentrations in the water column (Fig. 2) and the progressive sediment deposition after flow reduction. However, pesticide detection in flume sediments is influenced by factors such as hydrological characteristics and the physicochemical properties of compounds, including water solubility and sorption coefficients (Nilsson and Renöfält 2008; Khurshid et al. 2024). As a result, of the 96 pesticides analyzed (Table S2), 17 were detected above the LOQs in sediment samples under control and 21 under the low-flow treatment (Table S5). In the control, fungicides contributed 82%, herbicides 12%, and insecticides 5.9% to the total pesticide concentration in both treatments. Similarly, in the low-flow treatment, fungicides contributed to 71%, insecticides to 19%, and herbicides to 9.5% of the total pesticide concentration. The insecticides acetamiprid, thiacloprid, and indoxacarb, as well as the fungicide benalaxyl, were detected only in the low-flow treatment (Table S5). These differences in the detected pesticides between the two treatments can be explained by reduced flow velocity, higher residence time, and increased organic carbon content that were found in the low-flow treatment (Gaullier et al. 2020). The increased sedimentation of suspended solids contaminated with pesticides under low-flow conditions likely increased the exposure of aquatic insect larvae (e.g., Chironomidae) inhabiting the stream sediment (Kraus 2019; Gaullier et al. 2020; Roodt et al. 2023).

### Emerging Insects’ Response to Control and Low-Flow Conditions

**Fig. 4:**
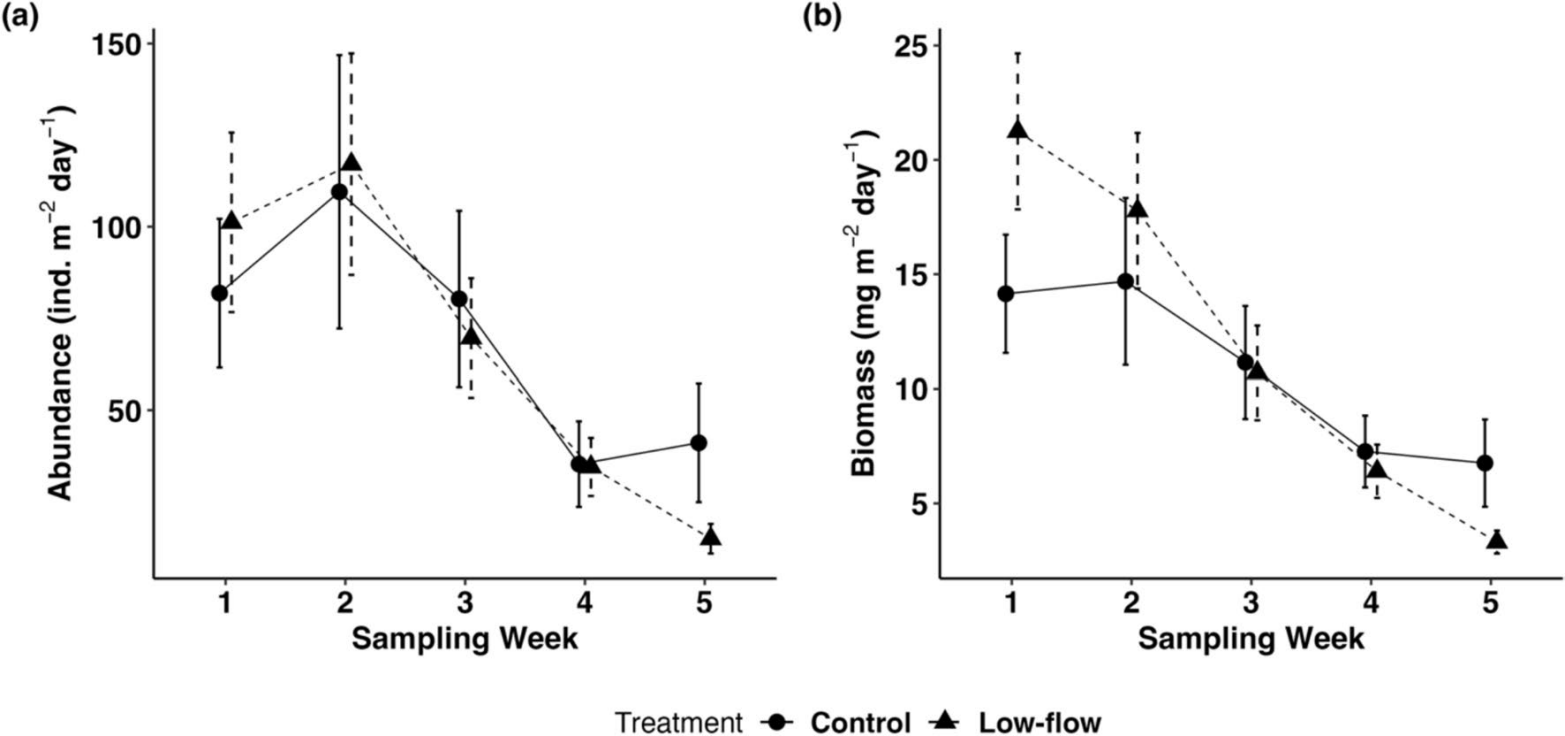
**a** Mean abundance (± standard error [SE]; n = 6) of emerging aquatic insects in the control (circles, solid lines) and low-flow treatments (triangles, dashed lines) across five sampling weeks. **b** Mean biomass (± standard error [SE]; n = 6) of emerging aquatic insects in the control and low-flow treatments across five sampling weeks

Total EPT abundance was significantly lower under the low-flow treatment, with a 2.9-fold reduction compared to the control (emmeans ratio = 2.94, SE = 1.54, *p* = 0.04, Table S7). This aligns with our expectation in hypothesis II, which predicted that sensitive taxa such as EPT would be more negatively affected by environmental stressors than more tolerant groups like Chironomidae. Indeed, most of these taxa are specialists of running, well-oxygenated waters, and have low tolerance to water pollution (Chadd et al. 2017; Tubić et al. 2024). They are often among the first groups to disappear during the drying of rivers affected by flow intermittence (Carlisle et al. 2014; Calapez et al. 2014; Tubić et al. 2024), a process that can be further exacerbated by pesticide accumulation in stream sediments, as observed in our experimental system. Trap position within each flume did not significantly influence total EPT abundance (ANOVA: χ^2^ = 4.4, *p* = 0.11), total emerging insect abundance (χ^2^ = 0.5, *p* = 0.78), or emerging insect biomass (χ^2^ = 1.3, *p* = 0.50). Furthermore, the abundance (emmeans ratio = 0.84, SE = 0.35, p = 0.67, Table S7) and biomass (emmeans ratio = 0.85, SE = 0.32, *p* = 0.66, Table S7) of total emerging aquatic insects were similar between the low-flow treatment and the control, contrary to our expectation of a decrease under low-flow conditions (hypothesis II). However, a significant decrease in total abundance (Fig. 4a, Table S6) and biomass (Fig. 4b, Table S6) was observed in weeks 4 and 5 compared to week 1, a pattern that was also evident in the control. This suggests that the temporal shift in emergence patterns is likely driven by natural seasonal phenology rather than experimental treatments, consistent with seasonal fluctuations in aquatic insect emergence observed in southwestern Germany (Ohler et al. 2023), particularly for dipterans (Chironomidae), the dominant group in the present study. Although the discharge rate in low-flow treatment was reduced by 60% compared to the control, the water in the low-flow flumes did not completely dry out. This partial reduction in flow may explain the lack of differences observed between the treatments (Dewson et al. 2007a). Additionally, Miller et al. (2007) suggested that an intensively managed agricultural catchment and the indirect effects of increased temperature and conductivity had a greater influence on aquatic insect abundance than low-flow conditions. In our study, the temperature and conductivity in the low-flow treatment did not differ substantially from the controls (Fig. S1, Table S1). Also, it is important to note that our study lasted only five weeks, which may not have been enough time to observe significant long-term effects (Dewson et al. 2007c).

**Fig. 5:**
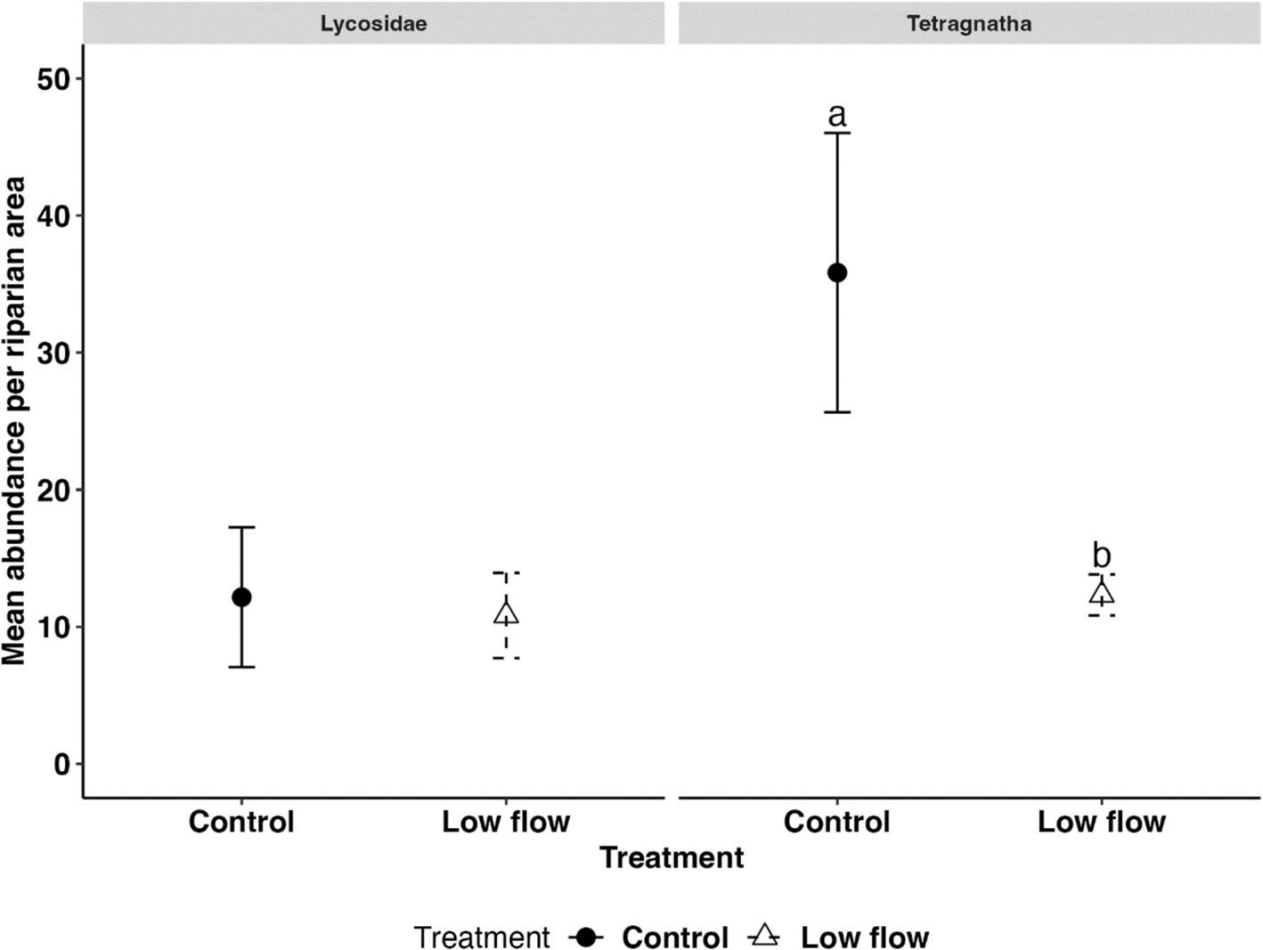
Mean abundance (n = 6) of two spider taxa in the control (circles, solid lines) and low-flow (triangles, dashed lines) treatments on day 39, with error bars representing the standard error (SE). Letters above the bars indicate significant differences (emmeans: *p* < 0.05)

### Riparian Spider Response to Control and Low-Flow Conditions

The long-jawed spiders (*Tetragnatha* sp.) and wolf spiders *Pardosa*, *Pirata*, and *Trochosa* from the Lycosidae family were the most abundant spider taxa found in the RSM riparian habitat.

Spider abundance did not differ between low-flow treatment and control (emmeans ratio = 1.6, SE = 0.557, *p* = 0.175, Table S7), contrary to hypothesis III, which predicted a decrease under low-flow conditions. However, when analyzed at the taxa level, we found a significant 2.6-fold reduction in the abundance of *Tetragnatha* sp. under low-flow conditions compared to the control (emmeans ratio = 2.58, SE = 0.919 *p* = 0.016, Table S7, Fig. 5). This is in accordance with our expectation that the effect would be more pronounced in spider species that specialized in preying on emerging aquatic insects. *Tetragnatha* sp., commonly found near freshwater habitat, constructs horizontal orb webs primarily in riparian vegetation to capture emerging insects (Roberts 1996; Bollinger et al. 2023). This species is particularly sensitive to changes in available resources and habitat structure (Kato et al. 2003; Baba and Tanaka 2016). Although we did not observe differences in total emerging insect abundance or biomass between treatments, the reduced abundance of *Tetragnatha* sp. could be explained by changes in habitat structure and habitat conditions (Kato et al. 2003; Baba and Tanaka 2016). For example, in our study, a 17-cm decrease in stream width was observed in flumes under low-flow conditions (Rovelli et al. 2024), creating drier stretches along the stream edges and likely affecting riparian vegetation. Similarly, Baba and Tanaka (2016) reported a reduction in the abundance of *Tetragnatha extensa* when the stream width narrowed to 50 cm. Furthermore, Baba and Tanaka (2016) noted that changes in vegetation around the stream edges contributed to a decline in *T. extensa* abundance. However, since vegetation–habitat-related responses to the low-flow treatment were not examined in our study, we cannot conclude that they were the primary cause of the observed reduction in *Tetragnatha* sp. abundance. Other factors, such as the significant decline in EPT taxa under low-flow conditions observed in this study, may have played an important role. In contrast, the ground-hunting Lycosidae showed no significant difference in abundance between control and low-flow treatment (Fig. 5). A possible explanation is their active hunting behavior and generalist strategy, which include the ability to switch prey types in response to changes in prey availability, allowing them to better adapt to altered habitat conditions (Kuusk and Ekbom 2012; Graf et al. 2020). Furthermore, differences in the sampling approaches used for the two spider taxa may have introduced sampling bias, so the absolute abundance effects should be interpreted with caution.

## Conclusion

Our study shows that low-flow conditions increased sediment pesticide concentrations over time, reduced EPT taxa abundance, and altered riparian spider communities. Notably, reduced EPT abundance and the response of *Tetragnatha* sp. to hydrological changes suggest potential cascading effects across the riparian food webs. Given the current widespread occurrence of flow cessation (Messager et al. 2021) and its projected intensification due to global change (Mimeau et al. 2024), the ecological response observed in this mesocosm study may become increasingly relevant. As riparian zones are ecologically important interfaces, these findings highlight the need to consider the broader and long-term effects of hydrological alterations in linked aquatic–terrestrial ecosystems.

## Supplementary Information

Below is the link to the electronic supplementary material.

Supplementary file1 (DOCX 356 KB)

Supplementary file2 (XLSX 51 KB)

## Data Availability

The datasets generated during and/or analyzed during the current study are available in the GitHub repository at https://github.com/colinog/pollution_flow.
