# Supplementary material for: Flow Reduction in a Pesticide-Exposed Stream Mesocosm Affects Emerging Aquatic Insects and Alters Riparian Spider Communities
Source: Arch Environ Contam Toxicol. 2025 Aug 15;89(2):125–35. doi: 10.1007/s00244-025-01146-5 (PMC12414100; doi:10.1007/s00244-025-01146-5)
Supplement: Supplementary file 1 — Supplementary file1 (DOCX 356 KB) [file 244_2025_1146_MOESM1_ESM.docx]

# Supplementary Information for

Title: **Flow reduction in a pesticide-exposed stream mesocosm affects emerging aquatic insects and alters riparian spider communities**

Collins Ogbeide^1, *^, Alessandro Manfrin^1^, Gemma Burgazzi^5^, Florian Burgis^1^, Anja Knäbel^1^, Sebastian Pietz^1^, Nina Röder^1^, Alexis Pieter Roodt^1^, Verena C. Schreiner^3,4^ Klaus Schwenk^1^, Mirco Bundschuh^1^, Ralf Schulz^1,2^

^1^ iES Landau, Institute for Environmental Sciences, RPTU Kaiserslautern-Landau, Fortstrasse 7, 76829 Landau, Germany

^2^ Eußerthal Ecosystem Research Station (EERES), RPTU Kaiserslautern-Landau, Birkenthalstraße 13, D-76857, Eußerthal, Germany

^3^ Faculty of Biology, University of Duisburg-Essen, Universitätsstrasse 2, 45141 Essen, Germany

^4^ Research Center One Health Ruhr, University Alliance Ruhr, Universitätsstrasse 2, 45141 Essen, Germany

^5^ ALPSTREAM Group, Department of Life Science and System Biology, University of Turin, Turin, Italy

*Correspondence to **c.ogbeide@rptu.de**

**Table S3** Size categories based on body length and shape for sorting and abundance estimation of emerging aquatic insects. Body length was measured from the anterior margin of the head (between the antennae) to the end of the posterior abdominal segment. Two body shapes (elongated and robust) were used for sorting.

| **Size category** | **Body length** | **Body shape** |
| --- | --- | --- |
| A | < 3 mm | Elongated |
| B | < 3 mm | Robust |
|  | 3–5 mm | Elongated |
| C | 3–5 mm | Robust |
|  | 5–8 mm | Elongated |
| D | 5–8 mm | Robust |

**
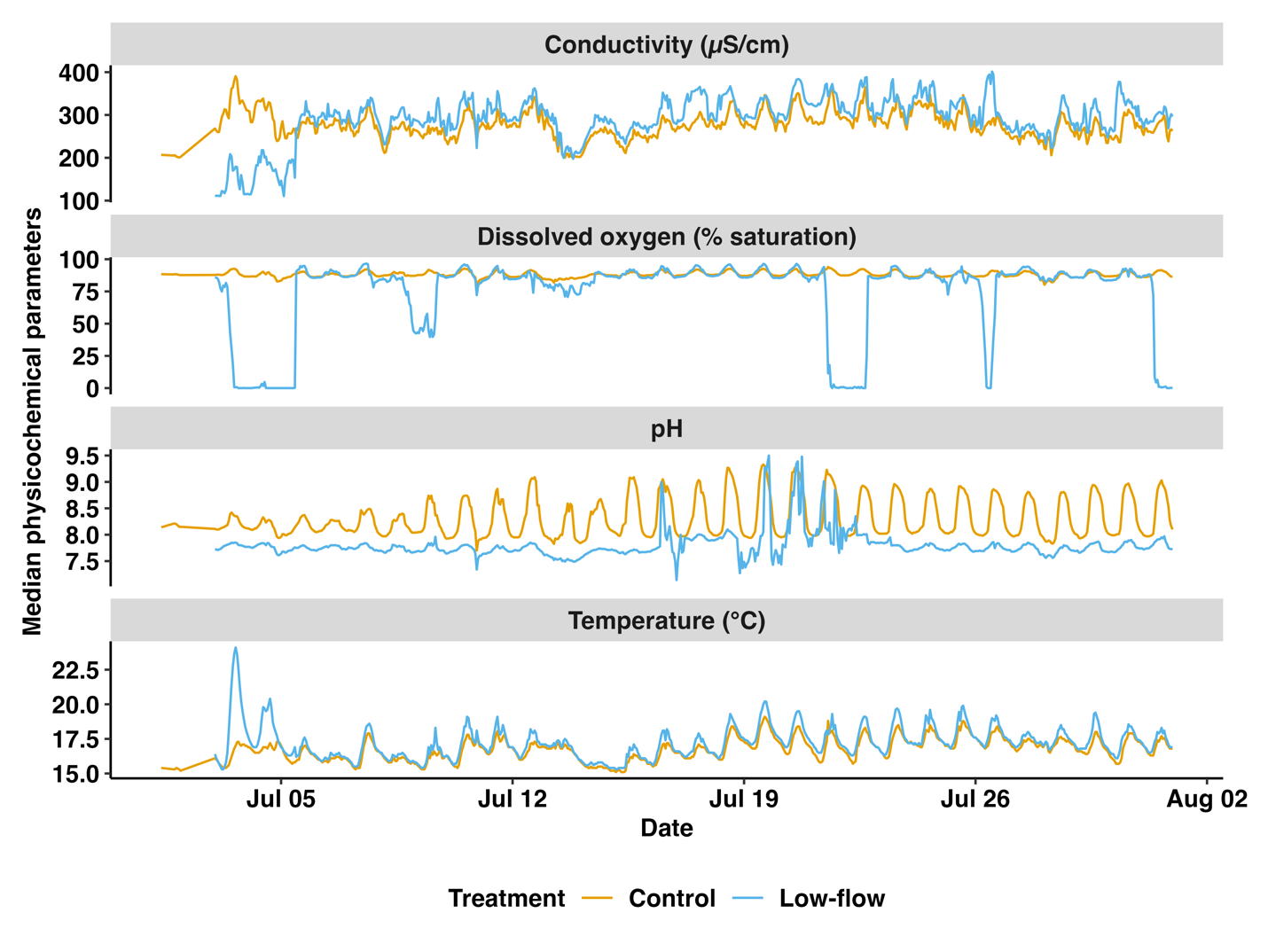
**

**Fig. S1** Time series plot of median hourly water physicochemical parameter measurements continuously logged using SENECT® probes during the treatment phase at the outlet section of the control and low-flow treatments flume. A temporary drop in dissolved oxygen (DO) values in low-flow treatment resulted from sensor coverage by sediment.

Table S8 Instrument parameters used for the HPLC-ESI-MS/MS analysis

| High performance liquid chromatography (HPLC) parameters | | |
| --- | --- | --- |
| Instrument: | Agilent 1260 Infinity II HPLC System | |
| Column: | ZORBAX Eclipse Plus C18 (3.0 ID x 150 mm, 2.7 micron) | |
| Eluent A: | H_2_O/MeOH (98:2), 0.1% Formic acid, 4 mM Ammonium formate | |
| Eluent B: | H_2_O/MeOH (2:98), 0.1% Formic acid, 4 mM Ammonium formate | |
| Injection volume for insect samples: | 10 µL | |
| Flow rate: | 0.45 mL/min | |
| Column temperature: | 45°C | |
| Elution gradient: | Time (min) | % Eluent A |
|  | 0 | 98 |
|  | 1 | 50 |
|  | 4 | 35 |
|  | 14 | 0 |
|  | 20 | 0 |
|  | 20.1 | 98 |
| Electrospray ionization (ESI) and triple quadrupole mass spectrometry (MS/MS) parameters | | |
| Instrument: | Agilent 6495 Triple Quadrupole Mass Spectrometer with an iFunnel Jet Stream ESI | |
| Capillary voltage: | 3000 V | |
| Nozzle voltage: | 0 V | |
| Gas flow: | 11 L/min | |
| Gas temperature: | 250 °C | |
| Sheath gas flow: | 12 L/min | |
| Sheath gas temperature: | 350 °C | |
| Nebulizer pressure: | 38 psi | |
| iFunnel High pressure RF | +/- 150 V | |
| iFunnel Low pressure RF | +/- 60 V | |
| Cycle time | 900 ms | |

**Supplementary tables (Excel file: Tables S1–2, S4–7, and S9)**

For additional table data, see Table S1–2, S4–7, and S9 in the supplementary Excel file “Ogbeide_et_al_SI_Statistical_summary_model_output.xlsx”
